# Exact distribution of a pattern in a set of random sequences generated by a Markov source: applications to biological data

**DOI:** 10.1186/1748-7188-5-15

**Published:** 2010-01-26

**Authors:** Gregory Nuel, Leslie Regad, Juliette Martin, Anne-Claude Camproux

**Affiliations:** 1 LSG, Laboratoire Statistique et Génome, CNRS UMR-8071, INRA UMR-1152, University of Evry, Evry, France; 2 CNRS, Paris, France; 3 MAP5, Department of Applied Mathematics, CNRS UMR-8145, University Paris Descartes, Paris, France; 4 EBGM, Equipe de Bioinformatique Génomique et Moleculaire, INSERM UMRS-726, University Paris Diderot, Paris, France; 5 MTi, Molecules Thérapeutique in silico, INSERM UMRS-973, University Paris Diderot, Paris, France; 6 MIG, Mathématique Informatique et Genome, INRA UR-1077, Jouy-en-Josas, France; 7 IBCP, Institut de Biologie et Chimie des Protéines, IFR 128, CNRS UMR 5086, University of Lyon 1, Lyon, France

## Abstract

**Background:**

In bioinformatics it is common to search for a pattern of interest in a potentially large set of rather short sequences (upstream gene regions, proteins, exons, etc.). Although many methodological approaches allow practitioners to compute the distribution of a pattern count in a random sequence generated by a Markov source, no specific developments have taken into account the counting of occurrences in a set of independent sequences. We aim to address this problem by deriving efficient approaches and algorithms to perform these computations both for low and high complexity patterns in the framework of homogeneous or heterogeneous Markov models.

**Results:**

The latest advances in the field allowed us to use a technique of optimal Markov chain embedding based on deterministic finite automata to introduce three innovative algorithms. Algorithm 1 is the only one able to deal with heterogeneous models. It also permits to avoid any product of convolution of the pattern distribution in individual sequences. When working with homogeneous models, Algorithm 2 yields a dramatic reduction in the complexity by taking advantage of previous computations to obtain moment generating functions efficiently. In the particular case of low or moderate complexity patterns, Algorithm 3 exploits power computation and binary decomposition to further reduce the time complexity to a logarithmic scale. All these algorithms and their relative interest in comparison with existing ones were then tested and discussed on a toy-example and three biological data sets: structural patterns in protein loop structures, PROSITE signatures in a bacterial proteome, and transcription factors in upstream gene regions. On these data sets, we also compared our exact approaches to the tempting approximation that consists in concatenating the sequences in the data set into a single sequence.

**Conclusions:**

Our algorithms prove to be effective and able to handle real data sets with multiple sequences, as well as biological patterns of interest, even when the latter display a high complexity (PROSITE signatures for example). In addition, these exact algorithms allow us to avoid the edge effect observed under the single sequence approximation, which leads to erroneous results, especially when the marginal distribution of the model displays a slow convergence toward the stationary distribution. We end up with a discussion on our method and on its potential improvements.

## Introduction

The availability of biological sequence data prior to any kinds of data is one of the major consequences of the revolution brought by high throughput biology. Large-scale DNA sequencing projects now routinely produce huge amounts of DNA sequences, and the protein sequences deduced from them. The number of completely sequenced genomes stored in the Genome Online Database [1] has already reached the impressive number of 2, 968. Currently, there are about 99 million DNA sequences in Genbank [2] and 8.6 million proteins in the UniProtKB/TrEMBL database [3]. Sequence analysis has become a major field of bioinformatics, and it is now natural to search for patterns (also called motifs) in biological sequences. Sequence patterns in biological sequences can have functional or structural implications such as promoter regions or transcription factor binding sites in DNA, or functional family signature in proteins. Because they are important for function or structure, such patterns are expected to be subject to positive or negative selection pressures during evolution, and consequently they appear more or less frequently than expected. This assumption has been used to search for exceptional words in a particular genome [4,5]. Another successful application of this approach is the identification of specific functional patterns: restriction sites [6], cross-over hotspot instigator sites [7], polyadenylation signals [8], etc. Obviously the results of such an approach strongly depend on the biological relevance of the data set used. A convenient way to discover these patterns is to build multiple sequence alignments, and look for conserved regions. This is done, for example, in the PROSITE database, a dictionary of functional signatures in protein sequences [9]. However, it is not always possible to produce a multiple sequence alignment.

In this paper, patterns refer to a finite family of words (or a regular expression), which is a slightly different notion from that of Position Specific Scoring Matrices (PSSM) [10] or in a similar way, from Position Weighted Matrices (PWM) or HMM profiles. Indeed, PSSM provide a scoring scheme to scan any sequence for possible occurrence of a given signal. When one defines a pattern ocurrence as a position where the PSSM score is above a given threshold, it is possible to associate a regular expression to this particular pattern. In that sense, PSSM may be seen as a particular case of the class of patterns we considered in this paper. However, this approach usually leads to huge regular expressions whose complexity grows geometrically with the PSSM length. For that reason, it seems far more efficient to deal with PSSM problems with methods and techniques that have been specifically developed for them [11,12].

Pattern statistics offer a convenient framework to treat non-aligned sequences, as well as assessing the statistical significance of patterns. It is also a way to discover putative functional patterns from whole genomes using statistical exceptionality. In their pioneer study, Karlin et al. investigated 4- and 6-palindromes in DNA sequences from a broad range of organisms, and found that these patterns had significantly low counts in bacteriophages, probably as a means of avoiding restriction enzyme cleavage by the host bacteria [6]. Then they analyzed the statistical over- or under-representation of short DNA patterns in herpes viruses using z-scores and Markov models, and used them to construct an evolutionary tree [4]. In another study, the authors analyzed the genome of *Bacillus subtilis *and found a large number of words of length up to 8 nucleotides with biased representation [5]. Another striking example of functional patterns with unusual frequency is the Chi motif (cross-over hot-spot instigator site) in *Escherichia coli *[7]. Pattern statistics have also been used to detect putative polyadenylation signals in yeast [8].

In general, patterns with unusual frequency are detected by comparing their observed frequency in the biological sequence data under study to their distribution in a background model whose parameters are derived from the data. Among a wide range of possible models, a popular choice consists in considering only homogeneous Markov models of fixed order. This choice is motivated both by the fact that the statistical properties of such models are well known, and that it is a very natural way to take into account the sequence bias in letters (order 0 Markov model), or words of size *h *≥ 2 (order *h *- 1 Markov model). However, it is well-known that biological sequences usually display high heterogeneity. Genome sequences, for example, are intrinsically heterogeneous, across genomes as well as between regions in the same genome [13]. In their study of the *Bacillus subtilis *chromosome, Nicolas et al. identified different compositional classes using a hidden Markov model [14]. These different compositional classes showed a good correspondence with coding and non-coding regions, horizontal gene transfer, hydrophobic protein coding regions and highly expressed genes. DNA heterogeneity is indeed used for gene prediction [15] and horizontal transfer detection [16]. Protein sequences also display sequence heterogeneity. For example, the amino-acid composition differs according to the secondary structure (alpha-helix, beta-strand and loop), and this property has also been used to predict the secondary structure from the amino-acid sequence using hidden Markov models [17]. In order to take into account this natural heterogeneity of biological data, it is common to assume either that the data are piecewise homogeneous (that is typically what is done with hidden Markov models [18]), or simply that the model changes continuously from one position to another (e. g., walking Markov models [19]). One should note that such fully heterogeneous models may also appear naturally as the consequences of a previous modeling attempt [20,21].

A biological pattern study usually first consists in gathering a data set of sequences sharing similar features (ribosome binding sites, related protein domains, donor or acceptor sites in eucaryotic DNA, secondary or tertiary structures of proteins, etc.). The resulting data set typically contains a large number of rather short sequences (ex: 5,000 sequences of lengths ranging between 20 and 300). Then one searches this data set for patterns that occur much more (or less) than expected under the null model. The goal of this paper is to provide efficient algorithms to assess the statistical significance of patterns both for low and high complexity patterns in sets of multiple sequences generated by homogeneous or heterogeneous Markov sources.

From the statistical point of view, studying the distribution of the random count of a simple or complex pattern in a multi-state homogeneous or heterogenous Markov chain is a difficult task. A lot of effort has gone into tackling this problem in the last fifty years with many concurrent approaches and here we give only a few references; see [22-25] for a more comprehensive review. Exact methods are based on a wide range of techniques like Markov chain embedding, moment generating functions, combinatorial methods, or exponential families [26-33]. There is also a wide range of asymptotic approximations, the most popular of which are Gaussian approximations [34-37], Poisson approximations [38-42] and Large Deviation approximations [43-45].

Recently several authors [46-49] have pointed out the connexion between the distribution of random pattern counts in Markov chains and the pattern matching theory. Thanks to these approaches, it is now possible to obtain an optimal Markov chain embedding of any pattern problem through minimal Deterministic Finite Automata (DFA).

In this paper, we first recall the technique of optimal Markov chain embedding for pattern problems and how it allows obtaining the distribution of a pattern count in the particular case when a single sequence is considered. We then extend this result to a set of several sequences and provide three efficient algorithms to cover the practical computation of the corresponding distribution, either for heterogeneous or homogeneous models, and patterns of various complexity. In the second part of the paper, we apply our methods to a simple but illustrative toy-example, and then consider three real-life biological applications: structural patterns in protein loop structures, PROSITE signatures in a bacteria proteome, and transcription factors in upstream gene regions. Finally, the results, methods and possible improvements are discussed.

## Methods

### Model and notations

Let (*X*_*i*_)_1≤*i*≤ℓ _be an order *d *≥ 0 Markov chain over the finite alphabet  (with cardinal || ≥ 2). For all 1 ≤ *i *≤ *j *≤ ℓ, we denote by  the subsequence between positions *i *and *j*. For all , *b *∈ , and 1 ≤ *i *≤ ℓ - *d*, let us denote by  the starting distribution and by  the transition probability towards *X*_*i*+*d*_.

Let  be a finite set of words (for simplification purpose, we assume that  contains no word of length less than *d *- in the general case, one may have to count the pattern occurrences already seen in , which results in a more complex starting distribution for our embedding Markov chain) over . We consider the random number *N*_ℓ _of matching positions of  in  defined by:(1)

where  is the set of all the suffixes of  and where  is the indicator function of event *A*.

### Overview of the Markov chain embedding

As suggested in [46-49], we perform an optimal Markov chain embedding of our pattern problem through a DFA. We use here the notations of [49]. Let (, , *σ*, ℱ, *δ*) be a *minimal *DFA that recognizes the language * of all texts over  ending with an occurrence of  where * denotes the set of all - possibly empty - texts over .  is a finite state space, *σ *∈  is the starting state, ℱ ⊂  is the subset of final states and  is the transition function. We recursively extend the definition of *δ *over  × * thanks to the relation  for all *p *∈ , *a *∈ , *w *∈ *. We additionally suppose that this automaton is non *d*-ambiguous (a DFA having this property is also called a *d*-th order DFA in [48]), which means that for all *q *∈ , the set  of sequences of length *d *that can lead to *q *is either a singleton or the empty set. A DFA is hence said to be non *d*-ambiguous if the past of order *d *is uniquely defined for all states. When the notation is not ambiguous, the set *δ*^-*d*^(*q*) may also denote its unique element (singleton case).

**Theorem 1**. We consider the random sequence over  defined by  and . Then  is a heterogeneous order 1 Markov chain over  such that, for all *p*, *q *∈ ' and 1 ≤ *i *≤ ℓ - *d *the starting distribution  and the transition matrix  are given by:(2)

And for all *i *≥ *d *we have:(4)

*Proof*. The result is immediate considering the properties of the DFA. See [48] or [49] for more details.   □

From now on, we will denote the cardinality of the set ' by *L *and call this the pattern complexity (even if technically, *L *depends both on the considered pattern and the Markov model order). A typical low complexity pattern corresponds to *L *≤ 50, moderate complexity to 50 <*L *< 100, and high complexity to *L *≥ 100.

**Proposition 2**. The moment generating function (*y*) of *N*_ℓ _is given by:(5)

where **1 **is a row vector of ones, and **1**^T ^denotes the transpose vector, and, for all 1 ≤ *i *≤ ℓ - *d*, **T**_*i*+*d *_= **P**_*i*+*d *_+ **Q**_*i*+*d *_with  and  for all *p*, *q *∈ *'*.

*Proof*. Since **Q**_*i*+*d *_contains all the counting transitions, we keep track of the number of occurrences by associating a dummy variable *y *to these transitions. Therefore, we just have to compute the marginal distribution at the end of the sequence and sum up the contribution of each state. See [46-49] for more details.   □

**Corollary 3**. In the particular case where (*X*_*i*_)_1≤*i*≤ℓ _is a homogeneous Markov chain, we can drop the indices in **P**_*i*+*d *_and **Q**_*i*+*d *_and Equation (5) is simplified into(6)

Corollary 3 can be found explicitly in [48] or [50] and its generalisation to a heterogeneous model (Proposition 2) is given in [51].

### Extension to a set of sequences

Let us now assume that we consider a set of *r *sequences. For any particular sequence *j *(with 1 ≤ *j *≤ *r*) we denote by ℓ_*j *_its length, by  its number of pattern occurrences, and by , , and  its corresponding Markov chain embedding parameters.

**Proposition 4**. If we denote by(7)

the moment generating function of , we have:(8)

**Corollary 5**. In the homogeneous case we get:(9)

### Single sequence approximation

Instead of computing the exact distribution of *N *= *N*_1 _+ ... + *N*_*r*_, which requires specific developments, one may study the number *N' *of pattern occurrences in a single sequence of length ℓ = ℓ_1 _+ ... + ℓ_*r *_resulting from the concatenation of our *r *sequences. The main advantage of this method is that we can rely on a wide range of classical techniques to compute the exact or approximated distribution of *N' *(Poisson approximation or large deviations for example).

The drawback of this approach is that *N *and *N' *are clearly two different random variables and that deriving the P-value of an observed event for *N *using the distribution of *N' *may produce erroneous results due to edge effects.

These effects may be caused by two distinct phenomena: forbidden positions and stationary assumption. Forbidden positions simply come from the fact that the artificial concatenated sequence may have pattern occurrences at positions that overlap two individual sequences. If we consider a pattern of length *h*, it is clear that there are *h *- 1 positions that overlap two sequences. It is hence natural to correct this effect by introducing an offset for each sequence, typically set to *h *- 1 for a pattern of length *h*. The length of our concatenated sequence has then to be adjusted to ℓ*' *= (ℓ_1 _- offset) + ... + (ℓ_r - 1_- offset) + ℓ_*r *_= ℓ - (*r *- 1) × offset. One should note that there is no canonical choice of offset for patterns of variable lengths.

Even if we take into account the forbidden overlapping positions with a proper choice of offset, there is a second phenomenon that may affect the quality of the single sequence approximation, and it is connected to the model itself. When one works with a single sequence, it is common to assume that the underlying model is stationary. This assumption is usually considered to be harmless since the marginal distribution of any non-stationary model converges very quickly towards its stationary distribution. As long as the time to convergence is negligible in comparison with the total length of the sequence, this approximation has a very small impact on the distribution. In the case where we consider a data set composed of a large number of relatively short sequences, this edge effect might however have huge consequences. This obviously depends both on the difference between the starting distribution of the sequences, and on the convergence rate toward the stationary distribution. This phenomenon is studied in detail in our applications.

### Algorithms

Let *n *be the observed number of occurrences of our pattern of interest. Our main objective is to compute both ℙ(*N *≤ *n*) and ℙ(*N *≥ *n*). We provide here various algorithms to perform these computations both for low or high complexity patterns, and for homogeneous or heterogenous models.

#### Heterogeneous case

**Algorithm 1**: Compute  (see Equation (10) for a proper definition of ) in the case of a heterogeneous model. The workspace complexity is *O*(*n *× *L*) and since all matrix vector products exploit the sparse structure of the matrices, the time complexity is *O*(ℓ × *n *× || × *L*) where || × *L *corresponds to the maximum number of non-zero terms in **T**_*i*+*d *_= **P**_*i*+*d *_+ **Q**_*i*+*d*_.

**Require: **The starting distributions  the matrices , , for all 1 ≤ *j *≤ *r*, 1 ≤ *i *≤ ℓ_*j *_- *d*, a *O*(*n *× *L*) workspace to keep the current values of **E**(*y*), and a dimension *L *polynomial row-vector of degree *n *+ 1.

   // *Initialization*

   **E**(*y*) ← **1**

   // *Loop on sequences*

   **for ***j *= 1, ..., *r ***do**

      **E**(*y*) ← (**E**(*y*)**1**^T^) × 

      // *Loop on positions within the sequence*

      **for ***i *= 1, ... ℓ_*j*_-*d ***do**

         

**Output: **return (*G*_*N *_(*y*)) = **E**(*y*)**1**^T^

When working with heterogeneous models, there is very little room for optimization in the computation of Equation (8). Indeed, since all terms  and  may differ for each combination of position *i *and sequence *j*, there is no choice but to compute the individual contribution of each of these combinations. This may be done recursively by taking advantage of the sparsity of matrices  and . Note that, so as to speed up the computation, it is not necessary to keep track of the polynomial terms of degrees greater than *n *+ 1. This may be done by using the polynomial truncation function  defined by(10)

This function also applies to vector or matrix polynomials. This approach results in Algorithm 1 whose time complexity is *O*(ℓ × *n *× || × *L*). In particular, one observes that the time complexity remains linear with *n*, which is a unique feature of this algorithm, while an individual computation of each (*y*) would obviously result in a final *O*(*r *× *n*^2^) complexity to perform the polynomial product . It is also interesting to point out that the number *r *of considered sequences does not appear explicitly in the complexity of Algorithm 1 but only through the total length .

#### Homogeneous case

**Algorithm 2**: Compute the (*G*_*N*_(*y*)) in the case of a homogeneous model. The workspace complexity is *O*(*n *× *L*) and since all matrix vector products exploit the sparse structure of the matrices, the time complexity to compute all ((*y*)) is *O*(ℓ_*r *_× *n *× || × *L*) where || × *L *corresponds to the maximum number of non-zero terms in **T **= **P **+ **Q**. The product updates of *U*(*y*) result in a additional time complexity of *O*(*r *× *n*^2^).

**Require: **The matrices **P **and **Q**, for all 1 ≤ *j *≤ *r*, the starting distributions , the length ℓ_*j *_(assuming ), a *O*(*n *× *L*) workspace to keep the current values of **E**(*y*) (a dimension *L *polynomial row-vector of degree *n *+ 1) and *U*(*y*) (a polynomial of degree *n *+ 1).

   // *Initialization*

   *U*(*y*) ← 1 and **E**(*y*) ← **1**

   // *Loop on sequences*

   **for ***j *= 1, ..., *r ***do**

      **for ***i *= 1, ..., ℓ_*j *_- ℓ_*j*-1 _**do**

         **E**(*y*)^T^← ((**P **+ *y***Q**)**E**(*y*)^T^)

      optionally return 

      

**Output: **return  (*G*_*N *_(*y*)) = *U *(*y*)

If we now consider a homogeneous model, we can dramatically speed up the computation of Equation (9) by recycling intermediate results in order to compute efficiently all (*y*). Without loss of generality, we assume that the sequences are ordered by increasing lengths: ℓ_1_≤ ...≤ ℓ_*r*_. If one stores the value of  in some polynomial vector **E**(*y*)^T^, it is clear that . By repeating this trick for all ℓ_*j*_, it is then possible to adapt Algorithm 1 to compute all  with a complexity *O*(ℓ_*r *_× *n *× || × *L*) (ℓ_*r *_being the length of the longest sequence), which is a dramatic improvement. Unfortunately, it is then necessary to compute the product , which results in a complexity *O*(*r *× *n*^2^) to get all polynomial terms of degree smaller that *n *+ 1 in *G*_*N*_(*y*). This additional complexity therefore limits the interest of this algorithm in comparison to Algorithm 1, especially when one observes a large number *n *of pattern occurrences. However, it is clear that Algorithm 2 remains the best option when considering a huge data set where we typically have ℓ_*r *_<< ℓ = ℓ_1 _+ ... + ℓ_*r*_.

#### Long sequences and low complexity pattern

**Algorithm 3**: Compute the (*G*_*N*_(*y*)) in the case of a homogeneous model using power computations. The workspace complexity is *O*(*n *× *K *× *L*^2^) with *K *= log_2_(max{ℓ_1_- *d*, ℓ_2 _- ℓ_1_, ..., ℓ_*r*_- ℓ_*r*-1_}). The precomputation time complexity is *O*(*n*^2 ^× *K *× *L*^3^). All ((*y*)) are computed with a total time complexity *O*(*r *× *n*^2 ^× *K *× *L*^3^). The product updates of *U*(*y*) result in an additional time complexity of *O*(*r *× *n*^2^).

**Require: **The matrices **P **and **Q**, for all 1 ≤ *j *≤ *r*, the starting distributions , the length ℓ_*j *_(assuming ), a *O*(*n *× *L*) workspace to keep the current values of **E**(*y*) (a dimension *L *polynomial row-vector of degree *n *+ 1) and *U*(*y*) (a polynomial of degree *n *+ 1), and a *O*(*n *× *K *× *L*^2^) workspace to store the values of (*y*) with 0 ≤ *k *≤ *K *= log_2_(max{ℓ_1 _- *d*, ℓ_2 _- ℓ_1_, ..., ℓ_*r *_- ℓ_*r*-1_}).

   // *Precompute all *(*y*)

    (*y*) ← **P **+ *y***Q**

   **for ***k *= 1, ..., *K ***do**

      

   // *Initialization*

   *U*(*y*) ← 1 and **E**(*y*) ← **1**

   // *Loop on sequences*

   **for ***j *= 1, ..., *r ***do**

      compute (*y*) using a binary decomposition and set **E**(*y*) ← ((*y*)**E**(*y*)^T^)

      optionally return 

      *U*(*y*) ← (*U*(*y*) × **E**(*y*)^**T**^)

**Output: **return (*G*_*N *_(*y*)) = *U *(*y*)

We now consider the case where ℓ_*r *_is large (ex: ℓ_*r *_= 100, 000 or 1, 000, 000 or more). With Algorithm 2, the time complexity is linear with ℓ_*r*_and may then result in an unacceptable running time. It is however possible to turn this into a logarithmic complexity by computing directly the powers of (**P **+ *y***Q**). This particular idea is not new in itself and has already been used in the context of pattern problems by several authors [50,51]. The novelty here is to apply this approach to a data set of multiple sequences.

If we denote by , it is clear that all (*y*) can be computed (and stored) for 0 ≤ *k *≤ *K *with a space complexity *O*(*n *× *K *× *L*^2^) and a time complexity *O*(*n*^2 ^× *K *× *L*^3^). It is therefore possible to compute all (*y*) using the same approach as in Algorithm 2 except that all recursive updates of **E**(*y*) are replaced by direct power computations. This results in Algorithm 3 whose total complexities are *O*(*n *× *K *× *L*^3^) in space and *O*(*r *× *n*^2 ^× *K *× *L*^3^) in time with *K *= log_2_(max{ℓ_1 _- *d*, ℓ_2 _- ℓ_1_, ..., ℓ_*r *_- ℓ_*r*-1_}). The key feature of this algorithm is that we have replaced ℓ_*r *_by the quantity *K*, which is typically dramatically smaller when we consider large ℓ_*r*_. The drawback of this approach is that the space complexity is now quadratic with the pattern complexity *L*, and that the time complexity is cubic with *L*. As a consequence, it is not suitable to use Algorithm 3 for a pattern of high complexity.

#### Long sequences and high complexity pattern

If we now consider a moderate or high complexity pattern, we cannot accept either a cubic complexity with *L *or even a quadratic complexity. Hence only Algorithms 1 or 2 are appropriate. However, if we assume that our data set contains at least one long sequence, it may be difficult to perform the computations. This is why we introduce an approach that allows computing *G*_*N *_(*y*) = **m**_*d*_(**P **+ *y***Q**)^ℓ-*d*^**1**^T ^for large ℓ and *L*. The technique is directly inspired from the partial recursion introduced in [51] to compute *g*(*y*) = **m**_*d*_(**P **+ **Q **+ *y***Q**)^ℓ-*d*^**1**^T^.

In this particular section, we assume that **P **is an irreducible and aperiodic matrix. We denote by *λ *the largest magnitude of the eigenvalues of **P**, and by *ν *the second largest magnitude of the eigenvalues of **P**/*λ*. For all *i *≥ 0 we consider the polynomial vector , where  and , and hence we have *G*_*N *_(*y*) = *λ*^ℓ-*d*^**m**_*d*_**F**_ℓ-*d*_(*y*).

Like in [51], the idea is then to recursively compute finite differences of **F**_*i*_(*y*) up to the point where these differences asymptotically converge at a rate related to *ν*^*i*^. We then derive an approximated expression for **F**_ℓ-*d*_(*y*) using only terms such as *i *≤ *α*. Unfortunately, this approach through partial recursion suffers the same numerical instabilities as in [51] when computations are performed in floating point arithmetic. For this reason, we chose here not to go further in that direction until a more extensive study has been conducted.

## Results and discussion

### Comparison with known algorithms

To the best of our knowledge, there is no record of any method that allows computing the distribution of a random pattern count in a set of heterogeneous Markov sequences. However, a great number of concurrent approaches exists to perform the computations for a single sequence, where the result for a set of sequences is obtained by convolutions.

For the heterogeneous case for a single sequence of length ℓ, any kind of Markov chain embedding techniques [48,52] may be used to get the expression of one (*y*) up to degree *n *+ 1 with complexity *O*(ℓ × *n *× || × *L*). In this respect, there is little novelty in Algorithm 1, except that it allows avoiding the *O*(*r *× *n*^2^) additional cost of the convolution product, which could be a great advantage. In the homogeneous case, the main interest of our approach is its ability to exploit the repeated nature of the data (a set of sequences) to save computational time. This is typically what it is done in Algorithm 2.

From now on, we will only consider the problem of computing the exact distribution of the pattern count *N*_ℓ _in a single (long) sequence of length ℓ generated by a homogeneous Markov source, and compare the novel approaches introduced in this paper to the most efficient methods available.

One of the most popular of these methods consists in considering the bivariate moment generating function(11)

where *y *and *z *are dummy variables. Thanks to Equation (6) it is easy to show that(12)

It is thus possible to extract the coefficients from *G*(*y*, *z*) using fast Taylor expansions. This interesting approach has been suggested by several authors including [46] or [48] and is often referred to as the "golden" approach for pattern problems. However, in order to apply this method, one should first use a Computer Algebra System (CAS) to perform the bivariate polynomial resolution of the linear system (**Id **- *z*(**P **+ *y***Q**)) **x**^T ^= **1**^T^. This may result in a complexity in *O*(*L*^3^) which is not suitable for high complexity patterns. Alternatively, one may rely on efficient linear algebra methods to solve sparse systems like the sparse LU decomposition. But the availability of such sophisticated approaches, especially when working with bivariate polynomials, is likely to be an issue.

Once the bivariate rational expression of *G*(*y*,*z*) is obtained, performing the Taylor expansions still requires a great deal of effort. This usually consists in first performing an expansion in *z *in order to get the moment generating function (*y*) of *N*_ℓ _for a particular length ℓ. The usual complexity for such task is *O*( × log ℓ) where *D*_*z *_is the denominator degree (in *z*) in *G*(*y, z*). In this case however, there is an additional cost due to the fact that these expansions have to be performed with polynomial (in *y*) coefficients. Finally, a second expansion (in *y*) is necessary to compute the desired distribution. Fortunately, this second expansion is done with constant coefficients. It nevertheless results in a complexity *O*( × log *n*) where *D*_*y *_is the degree of the denominator in (*y*) and *n *the observed number of occurrences.

In comparison, the direct computation of (*y*) = **m**_*d*_(**P **+ *y***Q**)**1**^T ^by binary decomposition (Algorithm 2) is much simpler to implement (relying only on floating point arithmetics) and is likely to be much more effective in practice.

Recently, [50] suggested to compute the full bulk of the exact distribution of *N*_ℓ _through Equation (6) using a power method like in Algorithm 3, with the noticeable difference that all polynomial products are performed using Fast Fourier Transforms (FFT). Using this approach, and a very careful implementation, one can compute the full distribution with a complexity *O*(*L*^3 ^× log_2 _ℓ × *n*_max _log_2 _*n*_max_) where *n*_max _is the maximum number of pattern occurrences in the sequence, which is better than Algorithm 3. There is however a critical drawback to using FFT polynomial products: the resulting coefficients are only known with an absolute precision equal to the largest one times the relative precision of floating point computations. As a consequence, the distribution is accurately computed in its center region, but not in its tails. Unfortunately, this is precisely the part of the distribution that matters for significant P-values, which are obviously the number one interest in pattern study. Finally, let us remark that the approach introduced by [50] is only suitable for low or moderate complexity patterns.

The new algorithms we introduce in this paper have the unique feature to be able to deal with a set of heterogeneous sequences. These algorithms, compared to the ones found in the literature, also display similar or better complexities. Last but not least, the approaches we introduce here only rely on simple linear algebra and are hence far easier to implement than their classical alternatives.

### Illustrative examples

In this part we consider several examples. We start with a simple toy-example for the purpose of illustrating the techniques, and we then consider three real biological applications.

#### A toy-example

**Figure 1:**
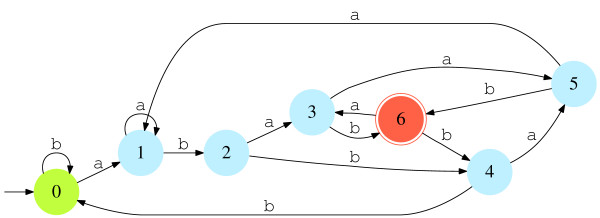
**Minimal DFA that recognizes the language ℒ = {a, b}* with  = {abab, abaab, abbab}**.

In this part we give a simple example to illustrate the techniques and algorithms presented above. We consider the pattern  = {abab, abaab, abbab} over the binary alphabet  = {a, b}. The minimal DFA that recognizes the language ℒ = * (which is the set of all texts over  ending with occurrence of ) is then given in Figure 1.

Let us now consider the following set of *r *= 3 sequences:

We process these sequences to the DFA of Figure 1 (starting each sequence in the initial state 0) to get the observed state sequences ,  and :

Therefore, Sequence *x*^1 ^contains *n*_1 _= 2 occurrences of the pattern (ending in positions 5 and 8), Sequence *x*^2 ^contains *n*_2 _= 1 occurrence (ending in position 5) and Sequence *x*^3 ^contains *n*_3 _= 1 occurrence (ending in position 8).

Let us now consider *X*^1^, *X*^2 ^and *X*^3^, three homogeneous order *d *= 1 Markov chains of respective lengths ℓ_1_, ℓ_2 _and ℓ_3 _such that *X*^1 ^and *X*^3 ^start with a, and *X*^2 ^starts with b, and the transition matrix of which is given by:

The corresponding state sequences ,  and  are hence order 1 homogeneous Markov chains defined over ' = {0, 1,2, 3, 4, 5, 6} with the starting distributions  =  = (0 1 0 0 0 0 0),  = (1 0 0 0 0 0 0) (since starting from 0 in the DFA of Figure 1, a leads to state 1 and b to state 0) and with the following transition matrix (please note that transitions belonging to **Q **are marked with a '*'. The others ones belong to **P**):

A direct application of Corollary 3 therefore gives (*y*) = 0.743104 + 0.208944*y *+ 0.0450490*y*^2 ^+ 0.0029030*y*^3 ^for the moment generating function of *N*_1 _(the number of pattern occurrences in *X*^1^);

(*y*) = 0.94816 + 0.05184*y *for the moment generating function of *N*_2 _(the number of pattern occurrences in *X*^2^); and (*y*) = 0.7761376 + 0.1880064*y *+ 0.0353376*y*^2 ^+ 0.0005184*y*^3 ^for the moment generating function of *N*_3 _(the number of pattern occurrences in *X*^3^). One should note that occurrences of  are strongly disfavored in Sequence *X*^2 ^since it starts with b. We then derive from these expressions the value of the moment generating function *G*_*N *_(*y*) of *N *= *N*_1 _+ *N*_2 _+ *N*_3_:(13)

Since we observe a total of *n *= *n*_1 _+ *n*_2 _+ *n*_3 _= 4 occurrences of Pattern , the P-value of over-representation is given by(14)

Let us finally compare the exact distribution of *N'*, the number of pattern occurrences over *X *= *X*_1_... *X*_ℓ _with ℓ = ℓ_1 _+ ℓ_2 _+ ℓ_3 _- 2 × offset, and a homogeneous order 1 Markov chain with transition matrix *π*:

As  contains both words of lengths 4 and 5, offset should be set either to 3 or 4. However, for both these values, 10^2 ^× ℙ(*N' *≥ 4) (either when *X*_1 _= a or when *X*_1 _= b) differs from the reference exact P-value 10^2 ^× ℙ(*N *≥ 4) = 0.333.

#### Structural motifs in protein loops

**Figure 2:**
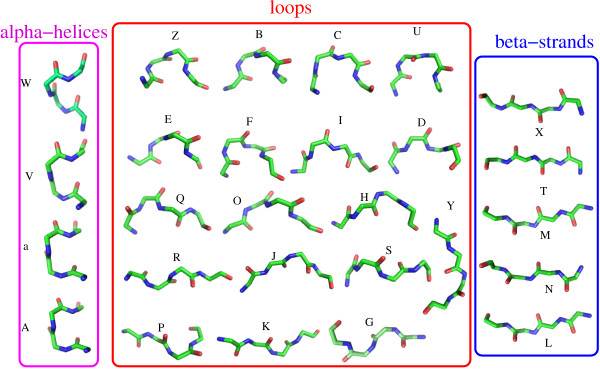
**Geometry of the 27 structural letters of the HMM-27 structural alphabet**.

Protein structures are classically described in terms of secondary structures: *α*-helices, *β*-strands and loops. Structural alphabets are an innovative tool that allows describing any three-dimensional (3D) structure by a succession of prototype structural fragments. We here use HMM-27, an alphabet composed of 27 structural letters (it consists in a set of average protein fragments of four residues, called structural letters, which is used to approximate the local backbone of protein structures through a HMM): 4 correspond to the alpha-helices, 5 to the beta-strands and the 18 remaining ones to the loops (see Figure 2) [53]. Each 3D structure of ℓ residues is encoded into a linear sequence of HMM-27 structural letters and results in a sequence of ℓ - 3 structural letters since each overlapping fragment of four consecutive residues corresponds to one structural letter.

We consider a set of 3D structures of proteins presenting less than 80% identity and convert them into sequences of structural letters. Like in [54], we then make the choice to focus only on the loop structures which are known to be the most variable ones, and hence the more challenging to study. The resulting loop structure data set is made of 78,799 sequences with length ranging from 4 to 127 structural letters.

In order to study the interest of the single sequence approximation described in the "Single sequence approximation" section, we first perform a simple experiment. We fit an order 1 homogeneous Markov model on the original data set, and then simulate a random data set with the same characteristics (loop lengths and starting structural letters). We then compute the z-score - these quantities are far easier to compute than the exact P-values and they are known to perform well for pattern problems as long as we consider events in the center of the distribution, and such events are precisely the ones expected to occur with a simulated data set - of the 77, 068 structural words of size 4 that we observe in the data, using simulated data sets under the single sequence approximation. We observe that high z-scores are strongly over-represented in the simulated data set: for example, we observed 264 z-scores of magnitude greater than 4, which is much larger than the expected number of 4.88 under *H*_0_. This observation clearly demonstrates that the single sequence approximation completely fails to capture the distribution of structural motifs in this data set. Indeed this experiment initially motivated the present work by putting emphasis on the need for taking into account fragmented structure of the data set.

We further investigate the edge effects in the data set by comparing the exact P-values obtained under the single sequence approximation. Table 1 gives the results for a selected set of 14 motifs whose occurrences range from 4 to 282. We can see that the single sequence approximation with an offset of 0 clearly differs from the exact value: e. g., Pattern ODZR has an exact P-value of 5.78 × 10^-5 ^and an approximate one of 2.81 × 10^-4^; Pattern BZOU has an exact P-value of 2.56 × 10^-11 ^and an approximate one of 4.49 × 10^-5^.

**Table 1:** P-values for structural patterns in protein loop structures using exact computations or the single sequence approximation (SSA) with offset or not.

Structural pattern	*n*	Exact	SSA (no offset)	SSA (offset = 3)
KYNH	16	1.62 × 10^-2^	5.95 × 10^-1^	8.43 × 10^-2^
PNKK	7	2.20 × 10^-2^	6.68 × 10^-2^	9.19 × 10^-3^
JLPQ	25	1.37 × 10^-3^	4.89 × 10^-1^	2.19 × 10^-2^
QYHB	110	1.71 × 10^-3^	9.46 × 10^-1^	2.59 × 10^-3^
ODZR	4	5.78 × 10^-5^	2.81 × 10^-4^	5.49 × 10^-5^
CPBQ	27	5.69 × 10^-6^	3.07 × 10^-3^	3.81 × 10^-6^
ZGBZ	50	3.45 × 10^-7^	4.84 × 10^-2^	9.71 × 10^-6^
BZOU	40	2.56 × 10^-11^	4.49 × 10^-5^	1.22 × 10^-9^
UOEI	52	5.74 × 10^-16^	1.96 × 10^-10^	2.30 × 10^-17^
EGZD	58	3.19 × 10^-32^	1.91 × 10^-23^	1.26 × 10^-32^
GIYC	149	1.05 × 10^-41^	1.06 × 10^-30^	3.85 × 10^-51^
DRPI	282	7.26 × 10^-167^	9.08 × 10^-174^	3.56 × 10^-222^

As explained in the Methods section, these differences may be caused by the overlapping positions in the artificial single sequence where the pattern cannot occur in the fragmented data set. Since we consider patterns of size 4, a canonical choice of offset is 4 - 1 = 3. We can see in Table 1 the effects of this correction. For most patterns, this approach improves the reliability of the approximations, even if we still see noticeable differences. For instance we get an approximated P-value larger than the exact one for Pattern BZOU, and an approximated P-value smaller than the exact one for Pattern UOEI. For other patterns, this correction is ineffective and gives even worse results than with an offset of 0. For example, Pattern DRPI has an exact P-value of 7.26 × 10^-167 ^and an approximate P-value with an offset of 3 equal to 3.56 × 10^-222^, while the approximation with no offset gives a P-value of 9.08 × 10^-174^.

Hence it is clear that the forbidden overlapping positions alone cannot explain the differences between the exact results and the single sequence approximation. Indeed, there is another source of edge effects which is connected to the background model. Since each sequence of the data set starts with a particular letter, the marginal distribution differs from the stationary one for a number of positions that depends on the spectral properties of the transition matrix. It is well known that the magnitude *μ *of the second eigenvalue of the transition matrix plays here a key role since the absolute difference between the marginal distribution at position *i *and the stationary distribution is *O*(*μ*^*i*^). In our example, *μ *= 0.33, which is very large, leads to a slow convergence toward the stationary distribution: we need at least 30 positions to observe a difference below machine precision between the two distributions. Such an effect is usually negligible for long sequences where 30 << ℓ, but is critical when considering a data set of multiple short sequences.

**Figure 3:**
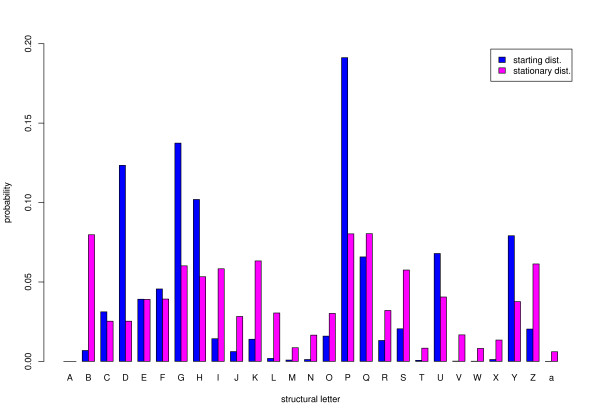
**Starting and stationary distributions of the 27 structural letters in the loop structure data set**.

However, this effect might be attenuated on the average if the distribution of the first letter in the data set is close to the stationary distribution. Figure 3 compares these two distributions. Unfortunately in the case of structural letters, there is a drastic difference between these distributions.

The example of structural motifs in protein loop structures illustrates the importance of explicitly taking into account the exact characteristics of the data set (number and lengths of sequences) when the single sequence approximation appears to be completely unreliable. As explained above, this may be due both to the great differences between the starting and the stationary distributions, as well as to a slow convergence and to the problem of forbidden positions.

#### PROSITE signatures in protein sequences

We consider the release 20.44 of PROSITE (03-Mar-2009) which encompasses 1, 313 different patterns described by regular expressions of various complexity [9]. PROSITE currently contains patterns and specific profiles for more than a thousand protein families or domains. Each of these signatures comes with documentation providing background information on the structure and function of these proteins. The shortest regular expression is for pattern PS00016: RGD, i. e., an exact succession of arginine, glycine and aspartate residues. This pattern is involved in cell adhesion. The longest regular expression, on the opposite, is for pattern PS00041:

[KRQ][LIVMA].(2)[GSTALIV]FYWPGDN.(2)[LIVMSA].(4, 9)[LIVMF].{PLH}[LIVMSTA][GSTACIL]GPKF.[GANQRF][LIVMFY].(4, 5)[LFY].(3)[FYIVA]{FYWHCM}{PGVI}.(2)[GSADENQKR].[NSTAPKL][PARL] (note that X means "any aminoacid", brackets denote a set of possible letters, braces a set of forbidden letters, and parentheses repetitions -fixed number of times or on a given range). This is the signature of the DNA-binding domain of the araC family of bacterial regulatory proteins.

**Figure 4:**
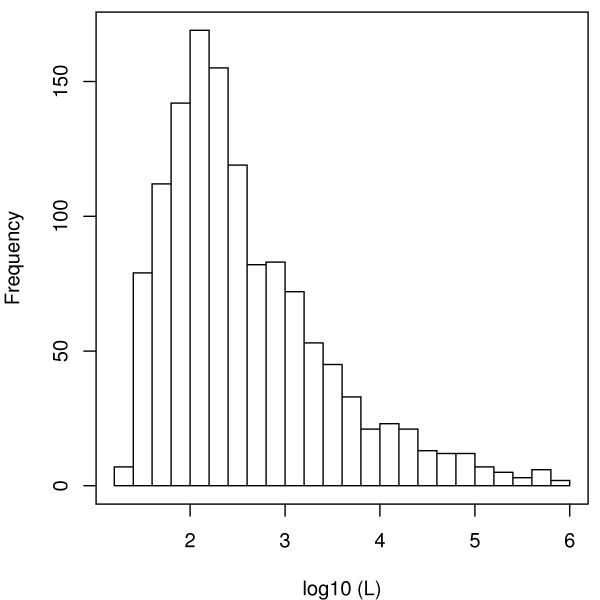
**Histogram of the log_10_(*L*) for 1, 276 PROSITE patterns in the framework of an order 1 Markov model**. Note that the 0.1% patterns with the largest complexities have been removed from the graph in order to improve readability.

This data set is useful to explore one of the key points of our optimal Markov chain embedding method using DFAs: the impact of the pattern complexity *L*. For this purpose, we first build 1-unambiguous (since we want to work with an order 1 Markov model) associated DFAs for 1,276 PROSITE patterns (37 patterns requiring a prohibiting computation time and/or memory were not computed). The repartition of the resulting pattern complexities is shown in Figure 4. There is a peak in the distribution at 2, meaning that many DFAs have ≃ 100 states. The smallest DFA is obtained for the RGD pattern (22 states), and the largest is for APPLE (PS00495) which is represented by the regular expression C.(3)[LIVMFY].(5)[LIVMFY].(3)[DENQ][LIVMFY].(10)C.(3)CT.(4)C.[LIVMFY]F.[FY].(13, 14)C.[LIVMFY][RK].[ST].(14, 15)SG.[ST][LIVMFY].(2)C which has 837, 507 states. The mean computing time of the DFA is 3 minutes, but 50% of the DFA could be computed in less than 0.01s, and 95% in less than 9s.

In Table 2, we can see that if short regular expressions usually lead to low complexity patterns, it is difficult to predict the result for longer regular expressions. For instance, the PROSITE signatures PUR_PYR_PR_TRANSFER and ADH_ZINC have the same size, but the former has a complexity of *L *= 102 while the latter has a complexity of *L *= 478. Indeed, we know from the theory of language and automata [55] that the minimal DFA corresponding to a regular expression of size *R *has a size *L *verifying *L *≤ 2^*R*^. Fortunately, in practice, *L *is usually dramatically smaller than this upper bound.

**Table 2:** Size of the regular expression (regex) and pattern complexity (*L*) for a selected subset of PROSITE signatures.

PROSITE signature	Accession number	pattern size	*L*
RGD	PS00016	3	22
ER_TARGET	PS00014	3	28
PPASE	PS00387	7	41
ALDEHYDE_DEHYDR_GLU	PS00687	8	44
PROKAR_NTER_METHYL	PS00409	21	46
GLY_RADICAL_1	PS00850	9	77
PEP_ENZYMES_PHOS_SITE	PS00370	12	96
PUR_PYR_PR_TRANSFER	PS00103	13	102

PILI_CHAPERONE	PS00635	18	226
SIGMA54_INTERACT_2	PS00676	16	313
EFACTOR_GTP	PS00301	16	320
ALDEHYDE_DEHYDR_CYS	PS00070	12	331
ADH_ZINC	PS00059	13	478
THIOLASE_1	PS00098	19	637
SUGAR_TRANSPORT_1	PS00216	15 to 17	796
FGGY_KINASES_2	PS00445	21 to 22	2668
PTS_EIIA_TYPE_2_HIS	PS00372	16	2758
MOLYBDOPTERIN_PROK_3	PS00551	27 to 28	3907
SUGAR_TRANSPORT_2	PS00217	26	6889

We now consider the complete proteome of the bacteria *Escherichia coli *(File NC_000913.faa, retrieved at ftp://ftp.ncbi.nih.gov/genomes/Bacteria/Escherichia_coli_K_12_substr__MG1655/). This data set encompasses a total of 4, 131 protein sequences with lengths ranging from 14 to 2, 358 aminoacids. We fit on this data set a homogeneous order 1 Markov model which is used to derive over-representation P-values of patterns.

Like for structural letters, we compare the exact P-values to the ones obtained using the single sequence approximation, see Table 3. Unlike in Table 1, we see here that the single sequence approximation performs already well with no offset, but that the use of the appropriate offset further improves this approximation.

**Table 3:** P-values for a selection of PROSITE patterns of low (or moderate) complexities using the complete proteome of *Escherichia coli *(NC_000913.faa).

PROSITE signature	*n*	Exact	SSA with no offset	SSA (offset)
RGD	215	5.35 × 10^-1^	5.91 × 10^-1^	5.55 × 10^-1^(2)
ER_TARGET	72	4.01 × 10^-2^	5.21 × 10^-2^	4.70 × 10^-2^(2)
PPASE	3	2.60 × 10^-2^	2.76 × 10^-2^	2.63 × 10^-2^(6)
ALDEHYDE_DEHYDR_GLU	12	1.99 × 10^-5^	2.41 × 10^-5^	1.95 × 10^-5^(7)
PROKAR_NTER_METHYL	10	6.79 × 10^-3^	8.01 × 10^-3^	5.10 × 10^-3^(20)
GLY_RADICAL_1	6	1.58 × 10^-6^	1.86 × 10^-6^	1.60 × 10^-6^(8)
PEP_ENZYMES_PHOS_SITE	4	1.49 × 10^-10^	1.74 × 10^-10^	1.49 × 10^-10^(12)
PUR_PYR_PR_TRANSFER	7	2.15 × 10^-14^	2.75 × 10^-14^	2.10 × 10^-14^(12)

This result is surprising, since, in this case, the starting distribution of the model strongly differs from the stationary distribution. Indeed, it is a biological fact that all protein sequences start with a methionine (M). As a consequence, it is hence clear that the starting distribution and the stationary distribution of the model strongly differ. This observation obviously does not favor the single sequence approximation. But in this example, this effect is corrected by the rapid convergence of the marginal distribution toward the stationary distribution ensured by a very low second magnitude eigenvalue of the matrix: *μ *= 0.049. We expect the same kind of behavior for the high complexity patterns of Table 4 but because of the numerical instabilities in the partial recursion approach suggested in the "Long sequences and high complexity pattern" section, unfortunately it was impossible to perform the computations for the single sequence approximation for such pattern in a reasonable time. However, it is possible to perform the exact computation for these high complexity patterns using Algorithm 2.

**Table 4:** Exact P-values for a selection of PROSITE patterns of high complexities using the complete proteome of *Escherichia coli *(NC_000913.faa). We use an order 1 homogeneous Markov model estimated over the data set.

PROSITE signature	*n*	Exact
PILI_CHAPERONE	10	3.27 × 10-^46^
SIGMA54_INTERACT × 2	12	1.58 × 10^-42^
EFACTOR_GTP	8	4.43 × 10^-20^
ALDEHYDE_DEHYDR_CYS	11	5.63 × 10^-9^
ADH_ZINC	12	8.93 × 10^-16^
THIOLASE_1	5	5.76 × 10^-9^
SUGAR_TRANSPORT_1	18	3.75 × 10^-8^
FGGY_KINASES_2	5	2.14 × 10^-4^
PTS_EIIA_TYPE_2_HIS	8	7.19 × 10^-19^
MOLYBDOPTERIN_PROK_3	11	2.59 × 10^-35^
SUGAR_TRANSPORT_2	10	1.22 × 10^-5^

Considering the multi-testing problem of this study (we consider a total of 1, 276 PROSITE signatures), we can set a significance threshold of 7.84 × 10^-7 ^at level 0.1% using a Bonferonni correction. Even at this stringent level, it is clear that many of the considered PROSITE signatures (2 out of 8 in Table 3, and 9 out of 11 in Table 4) are over-represented compared to our homogeneous order 1 Markov background model. However, this result is not a surprise since these patterns actually correspond to very precise functional signatures which are therefore expected to be strongly maintained through evolution in order keep their functional activities.

#### DNA motifs in gene upstream regions

Transcription factors regulate the expression of genes by activating or repressing the RNA polymerase. This is done by specific binding of the transcription factors (TFs) onto DNA, in proximity to the target genes, usually in the upstream regions. The transcription binding signatures on DNA are thus biologically important patterns.

We retrieved the sequence of transcription factor binding sites of *Saccharomyces cerevisiae *on the YEASTRACT website http://www.yeastract.com/consensuslist.php and searched for a subset of these transcription factor binding sites in the upstream regions of yeast genes, retrieved on the Regulatory Sequence Analysis Tools website [56]http://rsat.ulb.ac.be/rsat/. This data set comprises a total of 1,371 upstream sequences between positions -800 and -1 (the length is hence ℓ = 800 for each sequence).

On these data, we first fit an order 1 homogeneous Markov model. Since there is little difference between the starting distribution observed in the data set over  = {A, C, G, T}(0.30 0.16 0.19 0.35) and the stationary distribution (0.32 0.18 0.18 0.32), and since the magnitude of the second eigenvalue of the transition matrix is fairly low (*μ *= 0.092), we do not expect a great difference between the exact computations and the single sequence approximation. However, since exact computations are easily tractable, we do not further consider the single sequence approach for this particular problem.

We can see in Table 5 the P-values (column "homogeneous") of a selection of known TFs (marked with a star) as well as arbitrary candidate patterns. Several known TFs appear to be highly significant (e.g., TF AAGAAAAA with a P-value of 1.31 × 10^-99^) while others are not (e.g., TF WWWTTTGCTCR with a P-value of 4.15 × 10^-1^). It is the same for arbitrary candidate patterns. These results are difficult to interpret since these variations may be due either to statistical problems (e.g., insufficient Markov order) or real functional activities. Moreover, it is obviously impossible to distinguish a significant pattern which is a real TF of the organism from a significant pattern which is directly or indirectly implicated in another biochemical process.

**Table 5:** P-values for several DNA patterns (known transcription factors are marked with a star) in the upstream region data set.

DNA pattern	*n*	*L*	homogeneous	heterogeneous
CGCACCC*	28	10	2.95 × 10^-3^	3.74 × 10^-3^
AAGAAAAA*	427	11	1.31 × 10^-99^	1.29 × 10^-99^
AACAACAAC	25	10	1.76 × 10^-6^	1.38 × 10^-6^
TCCGTGGA*	22	11	1.12 × 10^-6^	1.55 × 10^-6^
GCGCGCGC	18	11	6.52 × 10^-10^	1.65 × 10^-9^
RTAAAYAA*	391	14	7.70 × 10^-12^	1.68 × 10^-12^
WWWTTTGCTCR*	15	17	4.15 × 10^-1^	4.09 × 10^-1^
AAAAAAAAAAAAAAAAAAAAAAAA	42	27	2.05 × 10^-23^	2.14 × 10^-22^
TAWWWWTAGM*	212	36	3.08 × 10^-9^	3.04 × 10^-9^
YCCNYTNRRCCGN*	11	40	3.10 × 10^-2^	3.05 × 10^-2^
GCGCNNNNNNGCGC	1	106	8.97 × 10^-1^	8.84 × 10^-1^
CGGNNNNNNNNCGG*	102	183	1.26 × 10^-14^	1.73 × 10^-13^
GCGCNNNNNNNNNNGCGC	6	464	2.88 × 10^-2^	2.84 × 10^-2^

**Figure 5:**
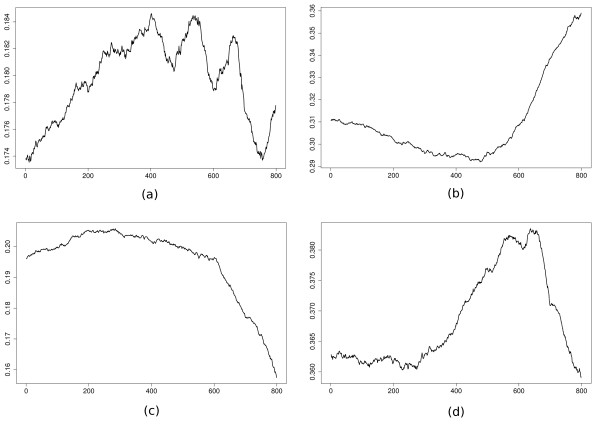
**Some transitions of the order 1 heterogeneous Markov model fitted using a sliding window of size 200 on the upstream region data set**. The plots respectively correspond to the following quantities: a) *π*_*i*_(A, G); b) *π*_*i*_(G, A); c) *π*_*i*_(G, G); d) *π*_*i*_(T, T), 1 ≤ *i *≤ 800.

**Figure 6:**
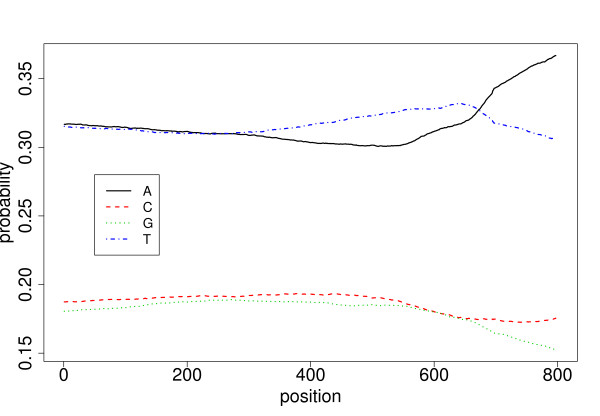
**Marginal distribution of the four nucleotides along the 800 positions of a upstream region**. The underlying model is an order 1 heterogeneous Markov model fitted using a sliding window of size 200 on the upstream region data set.

We now want to get rid of the homogeneous assumption of the model in an attempt to get a better fitting on the data. A simple way to achieve this is to perform a point-wise estimation of our transition function at position *i *by fitting the model on a window of size *w *centered around *i*. Small values of *w *lead to better fitting, while large values lead to better smoothing (resulting in a homogeneous model if *w *≥ ℓ, the length of the sequence). In this example, we achieve a satisfactory trade-off between the two extremes with an arbitrary choice of *w *= 200. We can see in Figure 5, that the model gives a unique profile for each transition probability (e.g., *π*_*i*_(A, G) or *π*_*i*_(G, G)), and these profiles are both quantitatively and qualitatively different from each other. In Figure 6 we consider the model in a more global way with the marginal distributions of the four nucleotides. According to this graph, it is clear that the upstream region has a bias in GC content that depends on the position. In particular, we observe a smaller GC content in the region [-200, -1] (positions 601 to 800) than in the region [-800, -201] (positions 1 to 599).

Thanks to Algorithm 1, it is possible to compute the P-values of DNA patterns in our heterogeneous model. The results are given in Table 5 (column "heterogeneous"). For most patterns, we can see that the P-values obtained with this heterogeneous model are in fact very close to the ones obtained with the homogeneous one. There are however several patterns for which a ratio factor of 10 may appear between these two P-values (e.g., Pattern GCGCGCGC or CGGNNNNNNNNCGG).

## Conclusion

In this paper, we introduce efficient algorithms to compute the exact distribution of random pattern counts in a set of multi-state sequences generated by a Markov source. These algorithms are able to deal both with low or high complexity patterns, and with either homogeneous or heterogenous Markov models.

This work, based on the recent notion of optimal Markov chain embedding through DFAs [46-49], is a natural extension of the methods and algorithms developed in [51] to obtain the first *k*^th ^moment of a random pattern count in one sequence. These computations of moments for a single sequence can easily be extended to a set of independent sequences by taking advantage of the fact that the cumulants (the first two cumulants are the expectation and the variance) of a sum of independent variables are the sum of the individual cumulants.

To the best of our knowledge, there currently exists no method specifically designed to compute the distribution of a random pattern count in a set of Markov sequences. However it exists a great deal of concurrent approaches to perform the computations for a single sequence, the result for a set of sequences being then obtained by convolution products. In this regard, Algorithm 1 has the interesting feature to completely avoid these convolutions and their possibly prohibitive *O*(*r *× *n*^2^) additional cost (*r *being the number of sequences in the data set, and *n *being the observed number of occurrences), especially for large *n*. Algorithm 1 also has the advantage to be able to deal both with heterogenous models and high complexity patterns. However, with a complexity in *O*(ℓ × *n *× || × *L*) (ℓ = ℓ_1 _+ ...+ ℓ_*r *_being the total length of the data set, *s *being the alphabet size, and *L *being the pattern complexity), this algorithm may be too slow when considering large data sets.

In the homogeneous model, Algorithm 2 can dramatically reduce the overall complexity by replacing ℓ by ℓ_*r *_the length of the longest sequence in the data set. Moreover this algorithm can deal with high complexity patterns, but this requires performing convolution products. However, it is clear that Algorithm 2 remains the best option when considering a data set with a large number of sequences with reasonable length: ℓ_*r *_<< ℓ = ℓ_1 _+ ... + ℓ_*r*_.

In the particular case where ℓ_*r *_is too high (e.g., ℓ_*r *_= 10^6 ^or more), it may be necessary to switch from linear to logarithmic complexity. This may be achieved by several methods. When dealing with low complexity patterns, the best known approach consists in computing the bivariate rational moment generating function *G*(*y, z*) of *N*_ℓ _the random number of pattern occurrences in a random sequence of length ℓ and then to perform fast Taylor expansions (logarithmic complexity) to get the probabilities of interest. However, this approach requires sophisticated computation in bivariate polynomial algebra, and has at least a cubic complexity with the denominator degree of the rational function *G*(*y*, *z*) whose value may be too high to perform the computations. Alternatively, the power approach proposed in Algorithm 3 also achieves logarithmic complexity, but with an easier implementation relying only on basic floating point linear algebra.

For high complexity patterns, the cubic complexity in *L *is prohibitive and prevents using neither power computations nor the plain formal inversion that is required to compute *G*(*y*, *z*). The partial recursion approach we introduce to deal with such a case appears to be a very interesting alternative, but its numerical instabilities in floating point arithmetic need to be further investigated. It is also possible to compute *G*(*y*, *z*) by solving the corresponding sparse linear system with appropriate sparse linear algebra methods (e.g., sparse LU), but the availability of such methods for multivariate polynomial matrices is likely to be an issue. Moreover, one should expect the denominator degree of the moment generating function to increase with the pattern complexity which could thus result again in untractable computations.

Another tempting option is to ignore the particular structure of the data set by approximating the distribution of *N *= *N*_1 _+... + *N*_*r *_by the one of *N'*, the random pattern count in a single sequence of length ℓ = ℓ_1 _+... +ℓ_*r*_. When one wants to use exact computations to get the distribution of *N'*, the resulting complexity is likely to be far greater that the one required to obtain the exact distribution of *N*. However, these approximations might be interesting if the distribution of *N' *is obtained through efficient asymptotic approximations like Poisson or Large Deviations approximations. Unfortunately, we have seen in our applications that this approach is subject to important edge effects, especially when the convergence of the marginal distribution of the model toward the stationary distribution is slow. It is therefore necessary to use this single sequence approximation with extreme caution when the stationary assumption of the model is clearly in contradiction with the observed data.

Thanks to Algorithm 1, it is possible for the first time (up to our knowledge) to study the distribution of patterns in a data set of upstream regions using an heterogeneous model. Despite the fact that there are some noticeable differences between this heterogeneous model and its homogeneous alternative, in practice we observe very little difference between the resulting P-values for most of the tested patterns. Some patterns are nevertheless more sensitive than others to the heterogeneity of the data, and their P-values may by altered by a factor 10 or more.

It should also be noted that heterogeneous Markov chains may be used to describe the behavior of homogeneous Markov chains under particular constraints. For example, this is exactly the distribution we get when considering the distribution of the hidden sequence of a HMM conditionally to the observed data (e.g., detection of CpG islands [20]). We get similar distribution when we take into account the special characters (N means "any nucleotides" in DNA sequences; X means "any aminoacid" in proteins) in biological sequences [21].

There are several interesting directions for further developments of this work. The first one could be to slightly change the statistic of interest for patterns problem by considering the *M *= *M*_1 _+ ... + *M*_*r *_number of matching sequences instead of the number of occurrences. Such a choice might be motivated by the nature of the selection pressure on a particular pattern: at least *k *occurrences of the pattern in a sequence insure a given biochemical activity (e.g., structured motifs in regulation [57]). In such a case, the pattern would match sequence *j *(*M*_*j *_= 1) if it occurs at least *k *times in the sequence, and would else mismatch the sequence (*M*_*j *_= 0). From a technical point of view, this is only a minor extension of the present work, where one only needs to adapt the existing method to get the moment generating function of each *M*_*j*_. However, the practical interest of such alternative statistic for pattern problem is yet to be studied.

A open problem remains open: how to deal with high complexity patterns (high *L*) in long homogeneous sequences (high ℓ)? The partial recursion we introduce here might be a solution, but it is necessary to study in further details its numerical stability issues. The only alternative seems to be the sparse LU bivariate polynomial approach suggested above to compute the bivariate moment generating function *G*(*y*, *z*). However, an exhaustive study of the relation between pattern complexity and the denominator degree of *G*(*y*, *z*) remains to be done in order to assess the practical interest of this approach.

Finally, let us point out that all the methods and algorithms presented in this paper are not yet available in an efficient implementation. One important task yet to be completed is to add these innovative techniques into the Statistics for Patterns package (SPatt) the purpose of which is to gather and make available the best pattern methods. SPatt is a C++ General Public License (GPL) program package which is freely available at the following url: http://stat.genopole.cnrs.fr/spatt

## Competing interests

The authors declare that they have no competing interests.

## Authors' contributions

GN developed the statistical results and algorithms and carried out their implementation and application with heterogeneous models. LR was in charge of the application to structural motifs in protein loops. JM was in charge of the PROSITE application and of the study of DNA upstream regions with homogeneous models. The redaction of the paper have been done by ACC and GN. All authors read an approved the final manuscript.
